# Effect of Simulated Cosmic Radiation on Cytomegalovirus Reactivation and Lytic Replication

**DOI:** 10.3390/ijms251910337

**Published:** 2024-09-26

**Authors:** Satish K. Mehta, Douglass M. Diak, Sara Bustos-Lopez, Mayra Nelman-Gonzalez, Xi Chen, Ianik Plante, Stephen J. Stray, Ritesh Tandon, Brian E. Crucian

**Affiliations:** 1 JES Tech, NASA, Johnson Space Center, Houston, TX 77058, USA; 2 Aegis Aerospace, Inc., Houston, TX 77598, USA; douglass.m.diak@nasa.gov; 3 Department of Health and Human Performance, University of Houston, Houston, TX 77004, USA; sara.bustoslopez@nasa.gov; 4 KBR, Houston, TX 77002, USA; mayra.a.nelman@nasa.gov (M.N.-G.); xi.chen@nasa.gov (X.C.); ianik.plante-1@nasa.gov (I.P.); 5 Department of Cell and Molecular Biology, University of Mississippi Medical Center, Jackson, MS 39216, USA; sstray@umc.edu (S.J.S.); rtandon3@gmail.com (R.T.); 6 NASA, Johnson Space Center, Houston, TX 77058, USA; brian.crucian-1@nasa.gov

**Keywords:** cytomegalovirus, radiation, galactic cosmic radiation, spaceflight, herpes viral reactivation

## Abstract

Human exploration of the solar system will expose crew members to galactic cosmic radiation (GCR), with a potential for adverse health effects. GCR particles (protons and ions) move at nearly the speed of light and easily penetrate space station walls, as well as the human body. Previously, we have shown reactivation of latent herpesviruses, including herpes simplex virus, Varicella zoster virus, Epstein–Barr virus, and cytomegalovirus (CMV), during stays at the International Space Station. Given the prevalence of latent CMV and the known propensity of space radiation to cause alterations in many cellular processes, we undertook this study to understand the role of GCR in reactivating latent CMV. Latently infected Kasumi cells with CMV were irradiated with ^137^Cs gamma rays, 150 MeV protons, 600 MeV/n carbon ions, 600 MeV/n iron ions, proton ions, and simulated GCR. The CMV copy number increased significantly in the cells exposed to radiation as compared with the non-irradiated controls. Viral genome sequencing did not reveal significant nucleotide differences among the compared groups. However, transcriptome analysis showed the upregulation of transcription of the UL49 ORF, implicating it in the switch from latent to lytic replication. These findings support our hypothesis that GCR may be a strong contributor to the reactivation of CMV infection seen in ISS crew members.

## 1. Introduction

Infection by cytomegalovirus (CMV), a member of the herpesvirus family, is highly prevalent in the human population, with estimated rates of infection ranging from 50% in developed countries to 90% in developing countries [1]. Herpes viruses of this nature have co-evolved with humans [2], and even more grandly, viruses in general have recently been regarded as integral and essential to the evolution of life on Earth [3]. This symbiosis is highlighted by the fact that humans become infected for life with these viruses, and primary infections are largely asymptomatic, although some immunocompetent patients may develop infectious mononucleosis. Regardless, all patients then become latently infected, meaning that the CMV genome is present in the cell but no virus is being shed and the transcriptional activity of the virus is limited to a few genes. Stress (acute and chronic alike) and/or an immunocompromised status, such as people living with Human Immunodeficiency Virus (HIV) or therapeutic immunosuppression, e.g., during hematopoietic stem cell transplant [HSCT], can initiate reactivation of latent CMV, resulting in conditions including retinitis, pneumonitis, and, more rarely, hepatitis, gastrointestinal ulceration, nephritis, and pancreatitis [1]. Additionally, active CMV replication during pregnancy can lead to intrauterine infection of the fetus, with the potential for congenital CMV, the consequences of which can include microcephaly, blindness, and developmental delay [4,5]. While anti-viral agents, like ganciclovir [6], are suitable prophylactics or treatments, no “cure” for latent viral infections exists. Thus, studying the cause and effect of reactivations as well as promoting non-reactivating countermeasures is key to limiting the virus’s detrimental possibilities.

Human space exploration presents a unique hostile environment difficult to replicate on Earth with hazards including microgravity, ionizing and non-ionizing cosmic radiation, physical and psychological stresses of traveling to and from space, altered diet and nutrition, continually interrupted sleep schedules, reduced protection from electromagnetic fields, and physical and social isolation. The synergistic effects of all these risks can trigger (or worsen) medical problems, potentially hindering performance during long-duration space missions [7]. Not surprisingly, these unique features of space exploration result in increased herpes viral (EBV, CMV, HSV1, and VZV) reactivation [2,8]. However, it is unclear whether these increases are due to a dysregulated immune system, one of the aforementioned features of spaceflight, or a combination with other factors. Previously, we reported that ionizing radiation (gamma, proton, carbon, and iron) increased EBV lytic gene transcription and reactivation in AKATA cells latently infected with EBV [9]. In this study, we focused on the effect of space radiation to study CMV latency and reactivation, genomics, and transcriptomics. However, not only was individual ion radiation studied, but also the newly available simulated galactic cosmic radiation (GCRsim) at Brookhaven National Laboratory.

The over-arching hypothesis is that modeled space radiation will induce reactivation and specific changes in the CMV genome/transcriptome. To accomplish this goal, CMV genomes and transcriptomes were harvested from mock-exposed vs. radiation (proton, carbon, iron, gamma, and GCRsim) exposed Kasumi-3 cells latently infected with CMV and sequenced using next-generation sequencing. Irradiated cells showed reactivation of CMV based on viral loading, but genome sequencing did not reveal significant nucleotide differences among the compared groups. Transcriptomics, however, revealed significant differences in expression levels of several genes. Most importantly, UL49 was significantly (*p* < 0.026) upregulated with a log Fold Change (logFC) of 1.48 in GCRsim-exposed Kasumi-3 cells compared with mock-exposed cells. Since UL49 is an essential subunit of the viral pre-initiation complex that regulates viral gene expression, it is possible that the GCRsim-mediated reactivation of CMV may be related to enhanced expression of UL49.

## 2. Results

**Figure 1:**
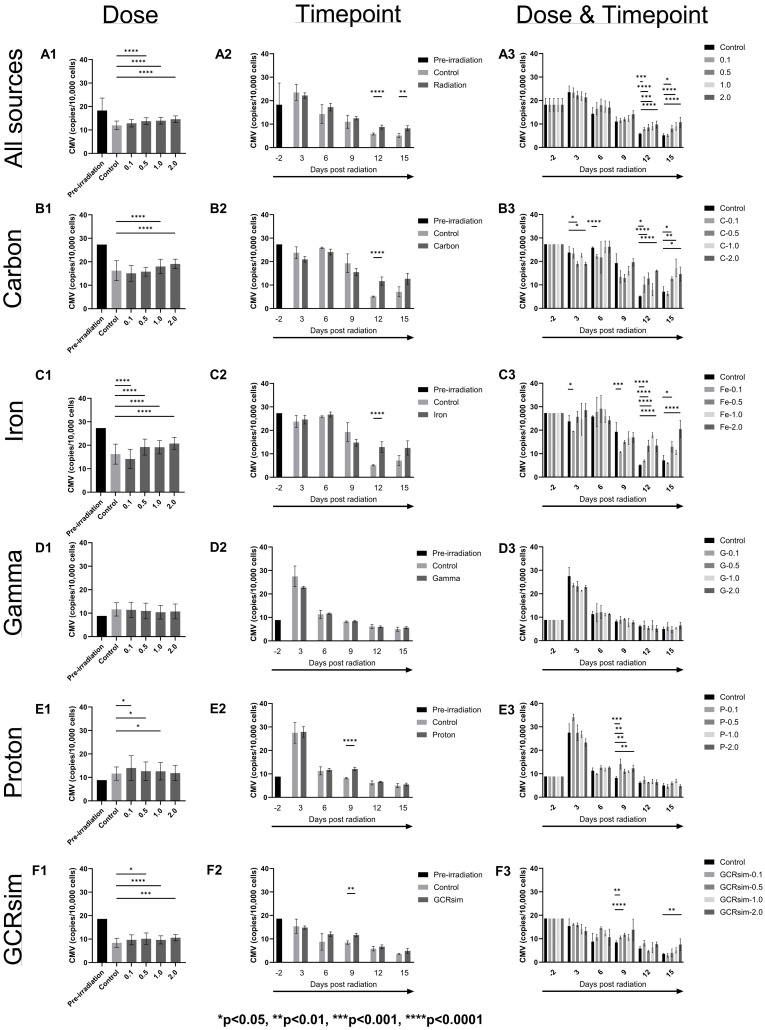
CMV viral load (copies/10,000 total cells) of CMV-infected Kasumi-3 cells separated by radiation source (**A**–**F**) and analyzed by dose (**column 1**), timepoint (**column 2**), and dose and timepoint (**column 3**). “All sources” in row (**A**) is a combined set of all the individual data sources to portray an overall picture of what radiation did to the infected Kasumi-3 cells, then separated by different types of individual radiations: carbon (**B**), iron (**C**), gamma (**D**), proton (**E**), and GCRsim (**F**). All data sets are compared with the controls (non-irradiated but traveled to and from BNL with the irradiated cells) within each graph. Significant differences (adjusted *p*-value) compared with the control group are defined above by the proper asterisk (*). Pre-irradiation values are included as a reference. All bars are mean ± SEM.

**Figure 2:**
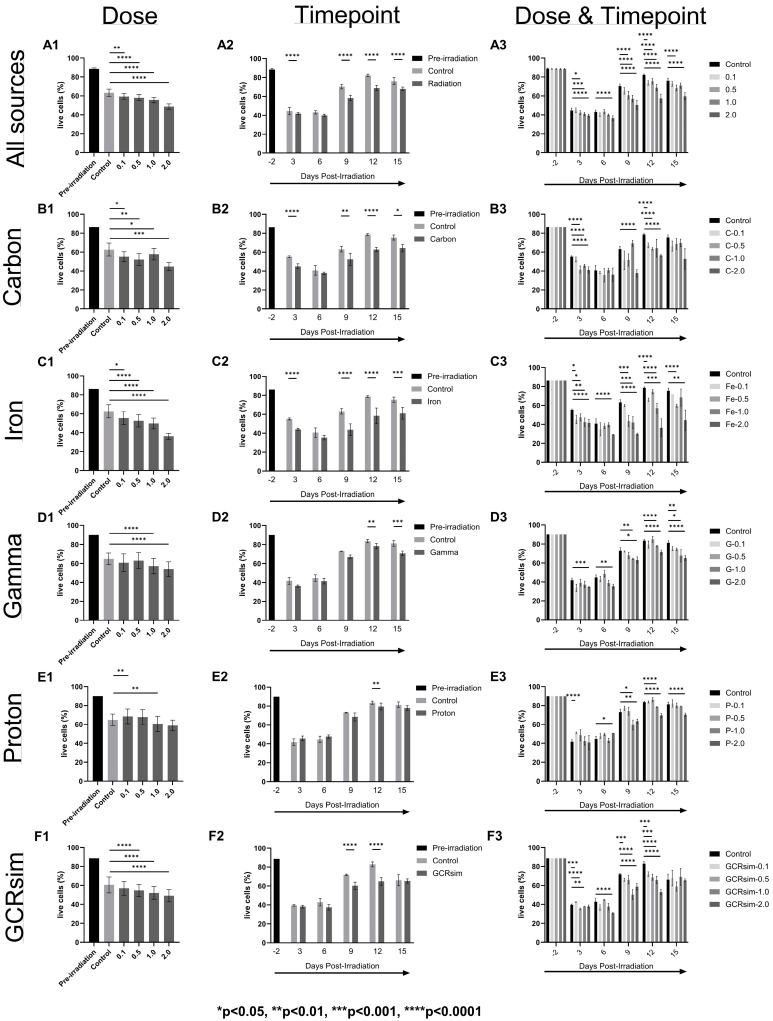
Cell viability (% live cells) of CMV-infected Kasumi-3 cells separated by radiation source (**A**–**F**) and analyzed by dose (**column 1**), timepoint (**column 2**), and dose and timepoint (**column 3**). “All sources” in row (**A**) is a combined set of all the individual data sources to portray an overall picture of what radiation did to the infected Kasumi-3 cells, then separated by different types of individual radiations: carbon (**B**), iron (**C**), gamma (**D**), proton (**E**), and GCRsim (**F**). All data sets are compared with the controls (non-irradiated but traveled to and from BNL with the irradiated cells) within each graph. Significant differences (adjusted *p*-value) compared with the control group are defined above by the proper asterisk (*). Pre-irradiation values are included as a reference. All bars are mean ± SEM.

**Figure 3:**
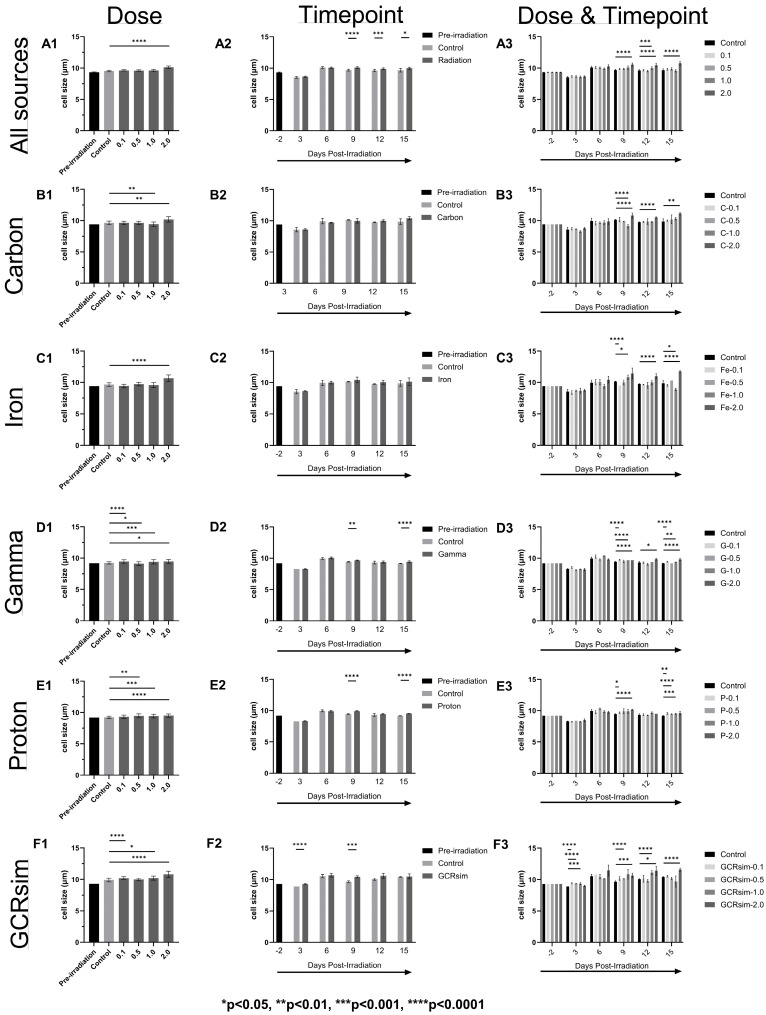
Cell size (µm) of CMV-infected Kasumi-3 cells separated by radiation source (**A**–**F**) and analyzed by dose (**column 1**), timepoint (**column 2**), and dose and timepoint (**column 3**). “All sources” in row (**A**) is a combined set of all the individual data sources to portray an overall picture of what radiation did to the infected Kasumi-3 cells, then separated by different types of individual radiations: carbon (**B**), iron (**C**), gamma (**D**), proton (**E**), and GCRsim (**F**). All data sets are compared with the controls (non-irradiated but traveled to and from BNL with the irradiated cells) within each graph. Significant differences (adjusted *p*-value) compared with the control group are defined above by the proper asterisk (*). Pre-irradiation values are included as a reference. All bars are mean ± SEM.

Figure 1, Figure 2 and Figure 3 present changes in the mean of CMV viral load in copies/10,000 cells (Figure 1), cell viability in live cell percentages (Figure 2), and cell size in µm (Figure 3) of CMV-infected Kasumi-3 cells before and after irradiation with carbon, iron, gamma, proton, and GCRsim.

### 2.1. Viral Load

Viral copy number is used to determine whether actual viral replication occurs from radiation as compared with the controls (non-irradiated cells but traveled to and from BNL with the irradiated cells). When analyzing all radiation sources together (Figure 1(A1)), 0.5, 1.0, and 2.0 Gy CMV viral loads were significantly higher (*p* < 0.001) as compared with controls throughout the entire experiment but also increased in the following order: control < 0.1 < 0.5 < 1.0 < 2.0. As shown in Figure 1(A1), the post-irradiation viral load in the CMV-infected Kasumi-3 cells decreased in controls as well as in all four radiation doses as compared with the pre-irradiation values (a separate set of the flasks for individual types of radiation but not irradiated). However, when the data were analyzed by timepoint (regardless of dose) (Figure 1(A2)), control and radiation both increased (non-significantly) post-radiation at Day 3 from an average of 18.27 to 23.53 and 22.21 copies/10,000 cells, respectively, and then tapered off as the experiment went on. Differences in viral load between control and radiation did not significantly occur until Day 12 (5.8 vs. 8.8 copies/10,000 cells, *p* < 0.001) and Day 15 (5.2 vs. 8.2 copies/10,000 cells, *p* < 0.01) (Figure 1(A2)). Within Day 12 and Day 15 (Figure 1(A3)), CMV viral load increased sequentially as dose increased, with all doses significantly different from control except for 0.1 Gy at Day 15.

When sorting out the data from each radiation source, proton and gamma showed an increase in CMV viral load at Day 3 post-irradiation compared with the pre-irradiation samples (Figure 1(D2,E2)). All other radiation types, carbon, iron, and GCRsim, showed a decrease at Day 3 post-irradiation from their pre-irradiation levels (Figure 1(B2,C2,F2)). However, when analyzing irradiated samples by dose (Figure 1-column 1) compared with the controls, all radiation sources (except gamma) showed significant increases of CMV viral load, with the most increases seen in iron ion radiation at 2.0 Gy (+4.50 CMV copies/10,000 cells, Figure 1(C1)) and carbon 2.0 Gy (+2.87 CMV copies/10,000 cells, Figure 1(B1)). As mentioned previously, when analyzed according to different time points post-irradiation (Figure 1-column 2), the viral load increased or was roughly the same as the pre-irradiation values and then started to decrease on Days 6, 9, 12, and 15 for all the radiation sources. Significant differences compared with controls were found in proton and GCRsim at Day 9 (*p* < 0.001) (Figure 1(E2,F2)) and carbon and iron at Day 12 (*p* < 0.001) (Figure 1(B2,C2)).

We also compared the results of viral load post each irradiation type and at each timepoint with their own respective controls (Figure 1-column 3). Viral load values were consistently higher post-radiation as compared with the control samples at each time point for all the experiments except for carbon and iron on Day 9 (Figure 1(B3,C3)) and gamma on Day 3 (Figure 1(D3)), which showed a decrease post-irradiation. A significant increase was observed 12 days post-irradiation for all sources as compared with their controls (Figure 1(A3)). For individual radiation types, carbon and iron showed an effect 3 days and beyond post-radiation (Figure 1(B3,C3)), while proton and GCRsim did not show any significant effect until 9 days post-irradiation (Figure 1(E3,F3)). Gamma did show an increasing trend, but this difference was not statistically significant (Figure 1(D3)).

### 2.2. Cell Viability

The cell viability was 86.3 to 89.9% before the cells were transported to NSRL (i.e., pre-irradiation values from each experiment). The trauma of transport negatively affected cell viability of Kasumi-3 cells infected with CMV, as evidenced by the substantial reduction in live cells in the control conditions (Figure 2). Radiation further significantly reduced the viability at all the dosages in an increasing order (control < 0.1 < 0.5 < 1.0 < 2.0) when compared with the controls for all sources (Figure 2(A1)). As represented in Figure 2(A2), the viability started to recover 9 days post-irradiation (controls: 70.25 ± 2.20%; radiation: 58.39 ± 2.12%) as compared with Day 3 (controls: 44.56 ± 2.57%; radiation: 41.89 ± 1.00%) and Day 6 (controls: 43.23 ± 1.86%; radiation: 39.96 ± 1.10%). The irradiated groups were significantly less viable than their corresponding controls at each timepoint (*p* < 0.001) except for Day 6 (Figure 2(A2)). The biggest fall was found at 2.0 Gy irradiation compared with controls at every timepoint analyzed (*p* < 0.001, Figure 2(A3)).

When sorting out each radiation source, proton and gamma showed the least reduction in viability compared with controls (max reduction at 2.0 Gy: proton = −5.94%, Figure 2(E1), and gamma = −10.93%, Figure 2(D1)). Carbon, iron, and GCRsim (except GCRsim 0.1 Gy, Figure 2(B1,C1,F1)) all showed significant decreases in cell viability compared with controls, with the most decreases seen in iron 2.0 Gy (−26.42%) and carbon 2.0 Gy (−17.81%). Proton and gamma radiation had significant reductions in viability at Day 12 (*p* < 0.01, Figure 2(D2,E2)), which were driven in large part by the higher dose radiations at 1.0 and 2.0 Gy (Figure 2(D3,E3)). Gamma radiation also had a significant reduction in viability at Day 15 (*p* < 0.001, Figure 2(D2)). Carbon, iron, and GCRsim all had significant reductions in viability at Days 9 and 12 (Figure 2(B2,C2,F2)), with the higher doses contributing to that the most (Figure 2(B3,C3,F3)). Carbon and iron also had reductions in viability at Days 3 and 15 (Figure 2(B2,C2)).

### 2.3. Cell Size

The normal size of Kasumi-3 cells infected with CMV is around 9.0 µm. In our data set, pre-irradiation cell size ranged from 9.2 to 9.4 µm (i.e., pre-irradiation values from each experiment). Post-irradiation saw a general increase in the size of these cells as compared with their respective controls for all the sources (Figure 3(A1)). However, a significant increase was only found at 2.0 Gy irradiation dose for all the sources (9.5 ± 0.1 to 10.1 ± 0.2; *p* < 0.001, Figure 3(A1)), but is evident by the fact that each individual radiation source had a significant cell size increase at 2.0 Gy (Figure 3(B1–E1)). For individual sources, iron showed a significant increase at 2.0 Gy (Figure 3(C1)), carbon showed at 1.0 and 2.0 Gy (Figure 3(B1)), gamma was significant at all doses (Figure 3(D1)), proton showed at 0.5, 1.0, and 2.0 Gy (Figure 3(E1)), and GCRsim showed at 0.1, 1.0, and 2.0 Gy (Figure 3(F1)), respectively. It was interesting to notice that this increase was observed 9 days post-irradiation for all sources (Figure 3(A2)) and at individual sources gamma and protons (Figure 3(D2,E2)). For post-GCRsim irradiation treatment, an increase in cell size was apparent even three days after treatment for doses of 0.1, 0.5, and 1.0 Gy (Figure 3(E2,E3)). Similar to the other individual radiation sources, GCRsim cell sizes also increased at Day 9 (Figure 3(E2)), which coincided with a dose of 2.0 Gy having statistical significance, which continued into Day 12s and 15 (Figure 3(E3)).

### 2.4. Genomics

Kasumi cells from the above experiments were subjected to genomic analysis in order to reveal any single nucleotide polymorphisms or other possible variations in the genomes of irradiated cells compared with mock. For this purpose, total nucleic acid (TNA) was extracted from the Kasumi-3 cells from the above experiment. The recovered TNA was subjected to DNA analysis. The same TNA was also used for transcriptional analysis described below. The initial set of 22 samples (pre-radiation, D6, D12, GCR, Proton) did not reveal statistically significant genomic variants upon comparison among the groups. This finding indicates that there were no stable genomic alterations induced by irradiation during this study.

### 2.5. Transcriptomics

The TNA recovered from this study was also examined for differences in transcriptional activity of the CMV genome in the irradiated and unirradiated groups. Data from the GCR-Day 6 cells compared with mock-exposed cells indicate that differential expression seems to be occurring, but these changes do not register as statistically significant (Supplemental Document S2). However, the UL49 sequence shows a significant (*p* < 0.026) increase in expression reflected as log Fold Change (logFC) of 1.48, summarized in Supplemental Document S1.

## 3. Discussion

Herpes viral reactivations in astronauts have generally been subclinical. However, medical events with viral etiology have occurred during spaceflight [10]. The cause of those reactivations, whether symptomatic or not, is not fully understood. Spaceflight imposes a multitude of stressors on the human body that can potentially directly (i.e., space radiation, microgravity) or indirectly (i.e., dysregulated immunity) cause the reactivation of herpes viruses. Our group has previously shown that lytic gene transcription of Epstein–Barr virus (EBV), which is consistently elevated in astronaut samples, is directly activated by a variety of radiation types and doses [9]. In the current study, we examined how ionizing radiation reactivated cytomegalovirus (CMV) in the Kasumi-3 myeloblast cell line. The GCR Simulator Project at NSRL generates an accelerator-based spectrum of energies that closely approximate those that are known to make up the shielded GCR environment in space, thus providing a unique Earthbound system to test CMV reactivation in culture for spaceflight applications.

In the current study, we tested the effect of carbon, iron, proton, gamma, and GCRsim individually across several doses (0.1, 0.5, 1.0, and 2.0 Gy) on CMV viral load, genomics, and transcriptomics. The CMV viral load in the infected Kasumi cells, taken as a whole, significantly increased in a dose-dependent manner, with increases seen at Day 3 at all the dosages tested, which tapered off through Day 15. However, at Days 12 and 15, the most significant viral load changes were seen at almost all doses compared with their corresponding controls. Surprisingly, however, carbon, iron, and GCRsim all had viral loads at Day 3 in controls and radiation groups that were either similar to or less than their respective pre-irradiation levels (Figure 1). Additionally, no radiation (at any dose) produced an increase in CMV at Day 3 or Day 6 compared with the control samples. In other words, if an increase in CMV viral load was seen early on for post-irradiation cells, it also occurred in the control flasks. We hypothesize that this may be attributed to the loss of cell viability resulting from cell transport from JSC to NSRL. Cells experience harsh conditions at the loading docks before and after shipping, including suboptimal temperature, humidity, and %CO_2_. Ideally, cell cultures would be undisturbed outside of experimental conditions, sample aliquoting, and media changes. However, with the NSRL facility located in New York, it was not feasible to do the many months-long pre-irradiation cell culture and infections outside our primary NASA facility at JSC. Based on our previous experiments using the latently infected EBV Akata cells, we did not anticipate such dampening of the experimental findings from the logistics of travel. We found that the cell viability decreased from 88.33% (before radiation) to 44.56% in controls and 41.89% in the irradiated groups (at Day 3 after radiation), accounting for a significant drop in the live cell count and, in turn, viral load. Even though the cell viability recovered after irradiation, the cell numbers never returned to the pre-irradiation levels. Even so, as the cells “settled down” and returned to the regulated physico-chemical conditions over time, differences between the controls and the radiation samples became apparent, as evidenced by the significant viral loads during the experimental timeline (particularly Days 12 and 15).

The most significant increase in the CMV load as compared with their corresponding controls was found at Day 9 for post-proton and GCRsim irradiations, at 12 days post-iron and carbon irradiations, and at Day 15 for carbon, iron, and GCRsim. On the contrary, our previous studies showed that gamma rays were more effective than protons, carbon ions, and iron ions in inducing latent virus reactivation, though these high-energy particles did induce more sustained and later reactivation of lytic gene transcription of EBV. Even though EBV and CMV are both herpesviruses, their replication and response mechanisms are expected to be different. Additionally, the Akata cell line (B-cell lymphoma) and the Kasumi-3 cell line (myeloblast leukemia) are different, and it may be that the Akata cells are just a more robust cell line and can handle perturbations in the cell culture conditions more easily. Not surprisingly, then, in normal human immunology, cells of the myeloid lineage generally have a shorter lifespan and faster turnover rate (days to weeks) than cells of the lymphoid system (weeks to months) [11,12]. Future studies should limit dyshomeostasis to the cell culture system or use a more stable model (in vivo or in vitro) to confirm these results.

In-depth correlation analysis of CMV genomes and transcriptomes before and after radiation exposure has the potential to identify specific loci or nucleotide changes due to radiation exposure. In the current study, viral genomic comparison of pre-radiation, Day 6, Day 12, GCRsim, and proton samples did not reveal significant differences; however, transcriptomic analysis revealed differentially expressed genes when comparing the GCRsim-Day6 cells with mock-exposed cells (Supplemental Document S2). Interestingly, only UL49 showed a significantly elevated level (LogFC 1.48, *p* value 0.026) in GCRsim-Day6 cells compared with mock. UL49 is an essential subunit of the viral pre-initiation complex that regulates viral gene expression [13]. UL49 displays leaky late expression kinetics and is localized to the nuclear replication compartments. UL49 serves as a fundamental HCMV effector that governs viral gene transcription required to complete the replication cycle. It is likely that the GCRsim-mediated reactivation of CMV may be related to over expression of UL49. Efforts to grow a UL49 virus in the laboratory were unsuccessful.

The radiation track structure likely plays an important role in CMV reactivation; however, this role is not clear. In general, the viral load is the highest 3 days post-irradiation and then decreases. The decay is the fastest for low-LET gamma rays and protons and slower for high-LET carbon and iron ions. The GCRsim follows the same trend, in-between low-LET and high-LET. We have also observed similar trends in our previous study on radiation-induced EBV reactivation [9]. It is possible that for a given dose, more of the DNA of the cell is affected by low-LET than high-LET radiation, which may favor viral reactivation. High-LET radiation tends to deposit energy in a more concentrated way than low-LET radiation. That leads to more damage to DNA and other structures, or more complex DNA damage, but the cell may die when the DNA damage is too complex to repair or when there is too much damage. Indeed, the viability of cells decreases similarly for most types of radiation 3 days post-irradiation. However, the viability of cells irradiated by protons and gamma recovers more than those irradiated by carbon and iron ions. Regarding cell size, the trends are harder to put into evidence. The changes in cell size observed may be due to the cytopathic effect on replication of HCMV. The cell size decreases slightly 3 days post-irradiation for all radiation types but returns (and even surpasses) the pre-irradiation size 6 days post-irradiation. The cell size increases the most with those irradiated with carbon and iron ions, especially at high doses. The cell size of those irradiated with GCRsim is similar to that of those irradiated by high-LET radiation.

To our knowledge, we have produced the only two studies that report the reactivation of herpesviruses (EBV and CMV) by spaceflight simulated radiation. However, medical research and cancer treatments have observed the reactivation of CMV by chemo- and radiotherapy for quite some time [14]. While reactivation is generally considered a by-product of reduced immune surveillance by an ablated immune system, case reports exist of radiotherapies causing suspected CMV reactivation and subsequent life-threatening pathologies [15]. The authors noted in their case report that the adverse effects (neurological deterioration) occurred during or shortly after radiation therapy and that successful administration of antiviral treatment greatly improved the surviving patients’ conditions. Additionally, CMV promoters, like IE-1, are used for innumerable studies to drive the expression of transgenes. A few studies have reported that radiation can induce activation of CMV promoters to overexpress transgenes of interest and thus enhance therapeutic delivery [16]. For example, Jung et al. in 2011 [17] found that radiation-induced expression of the CMV IEX-1 promoter (pIEX-TNF-α) increased TNF-α sensitization of cancer cells, leading to enhanced tumor regression in a xenograft model. These articles as well as our data presented here support the idea that increased radiation received through spaceflight may lead to direct reactivations of herpesviruses independent of the known mechanism of immune dysregulation.

## 4. Materials and Methods

### 4.1. Cell Culture

Kasumi-3 cells (hematopoietic stem cell-derived monocyte progenitor cells) (ATCC CRL-2725, ATCC, Manassas, VA, USA) were cultured in RPMI 1640 Medium (ATCC 30-2001) with 20% Fetal Bovine Serum (ATCC 30-2020), 20 U/mL penicillin, and 20 µg/mL Streptomycin. Cultures were maintained in nominal conditions at 37 °C, 95% humidity, and 5% CO_2_. To achieve the cell numbers needed for all the experimental conditions, primary frozen cell aliquots were subcultured for about 2 months prior to irradiation procedures.

### 4.2. Experimental Conditions

Kasumi-3 cells were infected with a low dose (multiplicity of infection [MOI] of 1) of CMV (TB40/E strain) and allowed to establish latency (10 days of culture with monitored viral gene expression). Once CMV latency was established, 500,000 cells/mL were seeded in 25-cm^2^ (T-25) flasks and filled to capacity (70 mL) for transport to the NASA Space Radiation Laboratory (NSRL) at the Brookhaven National Laboratory (BNL) in Long Island, New York. Cell size, viability, and CMV viral load were determined from a pooled sample prior to transport and were regarded as pre-irradiation baselines (Day 2). Filtered flask caps were replaced with plug caps, tightened, and parafilm wrapped. Transport was overnight (≈18 h) from Johnson Space Center (JSC) to BNL. Once received at BNL, plug caps were loosened and flasks were kept upright in nominal incubator conditions overnight. The next day (irradiation day), duplicate flasks of the cells were exposed to acute doses (0.1, 0.5, 1.0, and 2.0 Gy) within each modeled radiation type (gamma, proton, carbon, iron, and GCRsim) at room temperature. Control flasks of mock-irradiated cells were also included and placed out of the incubator during radiation treatment. After irradiation, flasks were maintained in nominal incubator conditions overnight until transport back to the Johnson Space Center Immunology and Virology Laboratory (Houston, TX, USA) for analysis. Cells were then aseptically split and harvested from each flask by removing 25 mL of cells and media on Day 3 post-irradiation and then every three days following (i.e., Day 3, 6, 9, 12, and 15). Except for Day 15 (the last day), harvested cells and media were replaced with 25 mL of fresh RPMI 1640 complete media. For each harvest, cells were split into 3 subsamples and then frozen for analysis of cell count and characterization, determination of CMV viral load, and next generation sequencing.

### 4.3. Irradiation Setup

At NSRL, the cells were exposed to an acute dose of radiation (0.1, 0.5, 1.0, and 2.0 Gy) or sham-irradiated at room temperature. Radiation doses of 5 Gy repeatedly killed all the cells, thus were not used. Irradiations were performed using ^13^ s gamma rays, individual ions (150 MeV protons, 600 MeV/n carbon, and 600 MeV/n iron ions), and the simulated Galactic Cosmic Rays (GCRsim) beam. The irradiation times for gamma rays are given in Table 1. The ions used for the ion irradiation are detailed in Table 2.

NSRL has a simulated full GCRsim field, which consists of 33 different ion beams, and a simplified GCRsim, composed of six ion beams (five different ions with protons at two different energies), with protons constituting most of the exposure and delivered first and last in the sequence [18]. For this experiment, the simplified GCRsim was used with the same total doses (10, 50, 100, and 200 cGy). The details of the beams, energies, dose rates, and irradiation times are given in Supplemental Document S1 (file Mehta NSRL 21B GCR 5-26-21 beam records Final).

**Figure 4:**
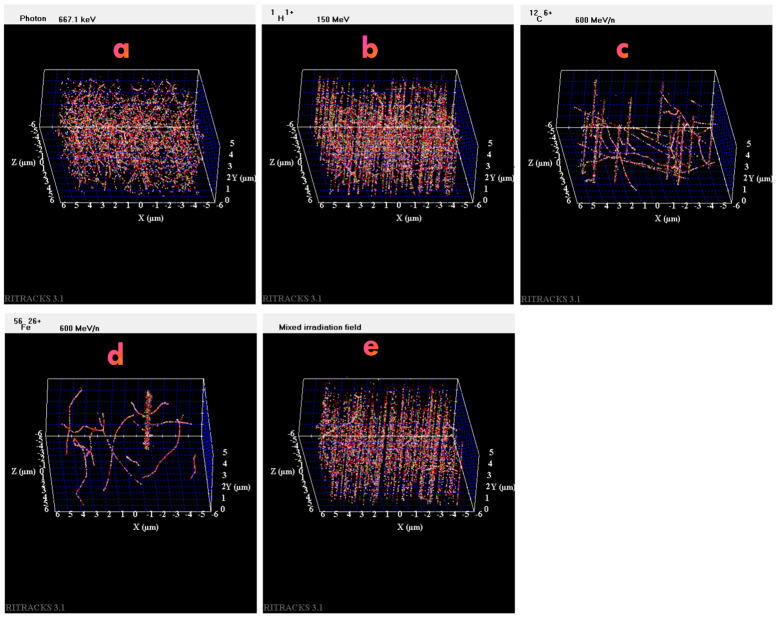
Simulation of the irradiation of a volume by (**a**) ^137^Cs photons, (**b**) 150 MeV protons, (**c**) 600 MeV/n carbon ions, (**d**) 600 MeV/n iron ions, and (**e**) simulated GCR mixed field. In all cases, the dosage to the irradiated volume is ~0.2 Gy.

At the scale of the Kasumi-3 cells, the track structures of the radiation used for this study differ greatly from one type to another. It might be useful to consider the radiation track structure to understand the differences between radiation types in viral reactivation. The simulation of an irradiated volume of 10 µm × 10 µm × 5 µm by gamma rays, protons, carbon ions, iron ions, and the simulated GCR by the Monte-Carlo simulation code RITRACKS [19] is shown in Figure 4. The details of these calculations, including the use of periodic boundary conditions, are described elsewhere [20,21]. The simulated volume is roughly the size of one Kasumi-3 cell. The dose to the volume is approximately 0.2 Gy in all cases. This dose was chosen to better illustrate the difference between the tracks of different types of radiation. In Figure 4a, electron tracks corresponding to compton and photoelectron effects are observed. In Figure 4b, 230 proton tracks are shown. The LET of the proton used for the simulation was 0.54 keV/µm. Therefore, this is often considered sparsely ionizing radiation (as opposed to high-LET radiations). However, the energy distribution and spatial configuration of the tracks are quite different for the same dose of gamma rays and protons. Tracks from higher LET ions are also shown. In Figure 4c, 14 tracks from 600 MeV/n carbon ions are shown. In Figure 4d, only one track for 600 MeV/n iron ions is sufficient to yield a dose of ~0.2 Gy to the volume. In Figure 4e, the simulation of the irradiation by the GCR field is shown. Most of the tracks are proton and helium tracks.

### 4.4. Cell Counts, Size, and Viability

Aliquots from each flask were taken at each experimental day (Day 2, 3, 6, 9, 12, and 15) and assessed for cell count (per mL), cell size (diameter in µm), and viability (percentage of live cells from total). Data were collected using a Cellometer Auto 2000 (Nexcelcom, Lawrence, MA, USA). In brief, a homogeneous sample of each cell culture flask was removed and mixed 1:1 with acridine orange and propidium iodide solution (AOPI) (Perkin Elmer, Shelton, CT, USA) and plated on Cellometer SD100 disposable slides for analysis [9].

### 4.5. Determination of CMV Viral Load

Frozen cells were thawed and pelleted by centrifugation at 14,000 rpm for 10 min. DNA was extracted from cell pellets with the Gentra Puregene Cell Kit (Qiagen, Valencia, CA, USA) using the manufacturer’s instructions. Total DNA recovered from extractions was quantified by a Qubit 2.0 Fluorometer and Invitrogen™ Quant-iT™ Qubit™ dsDNA HS Assay Kits (Invitrogen, Carlsbad, CA, USA). CMV DNA was quantified by Real-Time PCR (RT-PCR) using a QuanStudio 3 Real-Time PCR System (Applied Biosystems, Inc., Life technologies, Foster City, CA, USA). Each reaction mix (20 µL) contained 6.6 µL DNA and 13.4 µL of master mix (2X TaqMan Universal Master Mix, 830 nM IE-EX4 Forward, 830 nM IE-EX4 Reverse, 300 nM IE-EX4 Probe, 830 nM gB Forward, 830 nM gB Reverse, 300 nM gB Probe, and water). Primer and probe sequences were synthesized by IDT, Integrated DNA Technologies, Inc. (Coralville, IA, USA) and are listed in Table 3. Each sample was run in duplicate, and results were averaged. A housekeeping gene, GAPDH, was used for the qPCR assays. Viral load results (copies/µL) from the two duplicates were averaged and then normalized by the total cell count. For whole number simplification, values were multiplied by 10,000 to achieve the final variable (CMV copies/10,000 cells) used for statistical analysis and presentation in this manuscript.

### 4.6. Genomics and Transcriptomics

Commercial vendors were used for sequencing the genomes and RNA from exposed and control cells. Samples (cells) were shipped in frozen state on dry ice to the vendor for processing. Standard protocols for next-generation sequencing (NGS) of DNA and RNA were followed [22,23]. Briefly, total nucleic acid was extracted from the samples and assessed for purity using fluorometric methods. The DNA, or cDNA (for transcriptomics), was used for library preparation. Nucleotides were read on an Illumina sequencer at a read length and depth that was considered appropriate for the current purpose of discovering nucleotide changes and transcript abundance. Data were analyzed on the Illumina Connected Software (https://help.connected.illumina.com/, 20 September 2024) and obtained as sequence files as well as datasheets containing relative quantities of transcripts.

### 4.7. Statistical Method

Mixed models were applied to explore how CMV load, cell size, and viability change across days and different types of radiation and doses. To account for interactions between radiation type, dose, and days, mixed models with full three-way interaction terms were constructed. Random effects of flasks nested within radiation type were incorporated to address repeated measures. Robust standard errors were used to allow for non-homogeneous variance across time. The outcomes were assumed to follow a gamma distribution. Multiple comparisons between each group and the control group were adjusted using either simulation or the Bonferroni method. The alpha level was set at 0.05. All statistical analyses were performed using the software SAS 9.4. We conducted tests based on the mixed model described above. Specifically, we first compared each dose of radiation against the control group. Then, we compared the average of all radiation groups against the control group. Finally, we compared each dose against the control at each day post-radiation.

## 5. Conclusions

It is undeniable that herpesviruses reactivate from latency upon space travel. The cause of this reactivation is complicated and likely multifold. In this study on CMV (and our previous one with EBV), we show that space radiation has a direct effect on the reactivation of herpesviruses.

## Figures and Tables

**Table 1:** ^137^Cs gamma ray irradiation settings.

Dose (cGy)	Dose-Rate (cGy/Min)	Irradiation Time (Min)
10	132.52	0.08
50	132.52	0.38
100	132.52	0.75
200	132.52	1.51

**Table 2:** Individual ion irradiation settings used for the experiment.

Ion	Energy (MeV/n)	LET (keV/µm)	Dose (cGy)	Dose-Rate (cGy/Min)	Irradiation Time (Min)
^1^H^+^	150	0.54	10	22.25	0.45
^1^H^+^	150	0.54	50	56.86	1.08
^1^H^+^	150	0.54	100	60.33	1.89
^1^H^+^	150	0.54	200	65.62	3.24
^12^C^6+^	600	9.18	10	25	0.4
^12^C^6+^	600	9.18	50	25	2.0
^12^C^6+^	600	9.18	100	25	4.0
^12^C^6+^	600	9.18	200	25	8.0
^56^Fe^26+^	600	172.4	10	50	0.2
^56^Fe^26+^	600	172.4	20	50	1.0
^56^Fe^26+^	600	172.4	100	50	2.0
^56^Fe^26+^	600	172.4	200	50	4.0

**Table 3:** CMV primer and probe sequences for qPCR analysis.

Name	Sequence
IE-EX4 Forward	TCC CGC TTA TCC TCR GGT ACA
IE-EX4 Reverse	TGA GCC TTT CGA GGA SAT GAA
IE-EX4 Probe	TCT CAT ACA TGC TCT GCA TAG TTA GCC CAA TAC A
gB Forward	TGG GCG AGG ACA ACG AA
gB Reverse	TGA GGC TGG GAA GCT GAC AT
gB Probe	TGG GCA ACC ACC GCA CTG AGG

## Data Availability

Data supporting the reported results are available with the PI in the Immunology and Virology Laboratories of Johnson Space Center, NASA, Houston, Texas, USA. Please contact satish.k.mehta.@nasa.gov for any inquiries.

## Supplementary Materials

The following supporting information can be downloaded at: https://www.mdpi.com/article/10.3390/ijms251910337/s1.
