# Supplementary material for: Effect of Simulated Cosmic Radiation on Cytomegalovirus Reactivation and Lytic Replication
Source: Int J Mol Sci. 2024 Sep 26;25(19):10337. doi: 10.3390/ijms251910337 (PMC11477029; doi:10.3390/ijms251910337)
Supplement: Supplementary file 1 [file ijms-25-10337-s001.zip › ijms-3180070-supplementary.pdf]

Supplemental Document 1

Locustag	Description	logFC	PValue	FDR	PreD10B3RNA	D6GCR2D0-1
ABV71498.1	US7	0.25382348	0.69798057	0.99945292	3636.23491	4335.71699
ABV71499.1	US8	-0.2469002	0.70594332	0.99945292	2676.21903	2255.25924
ABV71500.1	US9	-0.0599634	0.92704635	0.99945292	2866.40291	2749.70607
ABV71501.1	US10	0.00052868	0.99945292	0.99945292	3777.06195	3778.44633
ABV71502.1	US11	0.09821772	0.88073967	0.99945292	2096.234	2243.91605
ABV71503.1	US12	0.14194872	0.82815365	0.99945292	4126.60273	4553.27359
ABV71504.1	US13	-0.0793468	0.90351451	0.99945292	2989.37389	2829.39927
ABV71505.1	US14	0.30111743	0.64527484	0.99945292	5334.75046	6572.94346
ABV71506.1	US15	0.52311638	0.42464091	0.99945292	6262.25483	8999.22314
ABV71507.1	US16	-0.0435204	0.94704097	0.99945292	3119.58836	3026.88715
ABV71508.1	US17	0.47924795	0.46432666	0.99945292	6003.67887	8369.23971
ABV71509.1	US18	-0.2302971	0.72493414	0.99945292	2200.00129	1875.40774
ABV71510.1	US19	0.13322106	0.83849715	0.99945292	9990.46529	10956.9417
ABV71511.1	US20	0.30062905	0.64581829	0.99945292	4983.52514	6138.1211
ABV71512.1	US21	0.05812018	0.92924705	0.99945292	3309.09842	3445.13099
ABV71513.1	US22	0.31132788	0.63410373	0.99945292	6951.73456	8626.06121
ABV71514.1	US23	0.91757366	0.1636482	0.99945292	7752.22508	14643.4791
ABV71515.1	US24	-0.1316522	0.84043408	0.99945292	5401.28955	4930.21659
ABV71516.1	US26	0.30502644	0.64091765	0.99945292	16729.6117	20668.4592
ABV71517.1	US27	0.15583112	0.81158294	0.99945292	8141.01551	9069.6091
ABV71518.1	US28	-0.2231973	0.73299036	0.99945292	3024.7491	2591.19223
ABV71519.1	US29	0.04803299	0.94146868	0.99945292	5558.45656	5746.63556
ABV71520.1	US30	-0.3025061	0.64393724	0.99945292	2777.79643	2252.35073
ABV71521.1	US31	-0.1857803	0.77685394	0.99945292	1053.67596	926.360677
ABV71522.1	US32	0.1090186	0.86793653	0.99945292	1102.19054	1188.70835

-

ABV71523.1	US33	0.59040082	0.36818076	0.99945292	1575.88148	2372.76308
ABV71524.1	US34	-0.0233923	0.97169578	0.99945292	1716.87697	1689.26305
ABV71525.1	US34A	0.02996448	0.96443665	0.99945292	400.245258	408.645762
ABV71526.1	RL1	0.01685978	0.97954357	0.99945292	2973.03391	3007.98183
ABV71527.1	RL10	-0.1928054	0.76835896	0.99945292	1855.00875	1622.949
ABV71528.1	RL11	0.27867001	0.67016633	0.99945292	2784.36611	3377.65354
ABV71529.1	RL12	-0.0858196	0.89555901	0.99945292	8627.84581	8129.57842
ABV71530.1	RL13/TRL14	0.00679756	0.99173187	0.99945292	11317.3727	11370.8228
ABV71531.1	UL1	0.09254016	0.8875002	0.99945292	3591.42631	3829.34526
ABV71532.1	UL2	-0.2568216	0.69725729	0.99945292	275.589749	230.644903
ABV71533.1	UL3	0.07978033	0.90299293	0.99945292	2571.77793	2718.0033
ABV71534.1	UL4	0.30296963	0.64321782	0.99945292	7086.66573	8742.69249
ABV71535.1	UL5	0.66477007	0.3111821	0.99945292	2974.88689	4716.15019
ABV71536.1	UL6	0.10988551	0.8665807	0.99945292	3595.13228	3879.6625
ABV71537.1	UL7	0.45866587	0.48369884	0.99945292	4168.88453	5729.18449
ABV71538.1	UL8	-0.2320504	0.7230923	0.99945292	1481.54758	1261.42112
ABV71539.1	UL9	-0.2804045	0.66827123	0.99945292	3149.57306	2593.22819
ABV71540.1	UL10	-0.0984379	0.88039738	0.99945292	3605.57639	3367.76461
ABV71541.1	UL11	0.09612515	0.883099	0.99945292	7528.01361	8046.68586
ABV71542.1	UL12	-0.214802	0.74359719	0.99945292	620.750748	534.875128
ABV71543.1	UL13	0.25748122	0.69373316	0.99945292	11992.5339	14335.7587
ABV71544.1	UL14	0.02022257	0.97541113	0.99945292	3781.77864	3835.16229
ABV71545.1	UL15A	-0.0324984	0.96077225	0.99945292	1026.89187	1004.01791
ABV71546.1	UL16	0.17356837	0.79071424	0.99945292	3357.10764	3786.29931
ABV71547.1	UL17	0.00886908	0.98936399	0.99945292	1958.43913	1970.51604
ABV71548.1	UL18	0.02358538	0.97125858	0.99945292	7028.04395	7143.88412
ABV71549.1	UL19	-0.1540934	0.81441411	0.99945292	768.315919	690.480454
ABV71550.1	UL20	-0.4104888	0.53066821	0.99945292	4822.98905	3628.65802
ABV71551.1	UL21A	0.29991507	0.64674987	0.99945292	2295.00901	2825.32735
ABV71552.1	UL22A	0.15067077	0.81795379	0.99945292	1729.51097	1919.90795
ABV71552.1	UL22A	0.15067077	0.81795379	0.99945292	1729.51097	1919.90795
ABV71553.1	UL23	-0.0374589	0.95431865	0.99945292	11169.639	10883.3564
ABV71554.1	UL24	0.01901242	0.9768886	0.99945292	3760.55351	3810.43994
ABV71555.1	UL25	0.04478193	0.94543076	0.99945292	5382.92813	5552.63789
ABV71556.1	UL26	-0.0909469	0.88965404	0.99945292	1517.5966	1424.87942
ABV71557.1	UL27	0.5493338	0.40191672	0.99945292	6963.35785	10190.2583
ABV71558.1	UL28	-0.2830408	0.66545022	0.99945292	2238.74557	1839.92391
ABV71559.1	UL29	-0.2364406	0.71788974	0.99945292	2332.74257	1980.11412
ABV71560.1	UL30	-0.0966033	0.88286162	0.99945292	1376.76957	1287.59771
ABV71561.1	UL31	-0.1126266	0.86324305	0.99945292	5859.14586	5419.13725

ABV71562.1	UL32	0.01060111	0.98709141	0.99945292	10130.9554	10205.6734
ABV71563.1	UL33	-0.016201	0.98029869	0.99945292	5216.49618	5158.24383
ABV71564.1	UL34	0.05451686	0.93357168	0.99945292	5900.41695	6127.65047
ABV71565.1	UL35	-0.0653556	0.92039816	0.99945292	6286.84903	6008.40152
ABV71566.1	UL36	-0.3907858	0.55052476	0.99945292	4719.05331	3599.28206
ABV71566.1	UL36	-0.3907858	0.55052476	0.99945292	4719.05331	3599.28206
ABV71567.1	UL37	0.37861307	0.56296065	0.99945292	3631.34976	4721.09466
ABV71567.1	UL37	0.37861307	0.56296065	0.99945292	3631.34976	4721.09466
ABV71567.1	UL37	0.37861307	0.56296065	0.99945292	3631.34976	4721.09466
ABV71568.1	UL38	0.19242532	0.76858788	0.99945292	3540.04802	4045.15676
ABV71569.1	vMIA	-1.3988604	0.08540709	0.99945292	10.7810171	4.07191506
ABV71570.1	UL40	-0.4426327	0.49932593	0.99945292	2265.0243	1666.57666
ABV71571.1	UL41A	-0.2169311	0.74082636	0.99945292	849.84736	731.199605
ABV71572.1	UL42	0.28572998	0.66263004	0.99945292	1195.34527	1457.16389
ABV71573.1	UL43	0.19018876	0.771184	0.99945292	3973.31015	4533.20487
ABV71574.1	UL44	-0.0538	0.93446995	0.99945292	4907.72111	4728.07509
ABV71575.1	UL44	0.22194544	0.73420813	0.99945292	27238.5765	31768.4996
ABV71576.1	UL46	1.04631246	0.11307996	0.99945292	6037.70646	12469.3673
ABV71577.1	UL47	-0.1103851	0.86589818	0.99945292	10078.398	9336.02868
ABV71578.1	UL48	0.17500613	0.78887776	0.99945292	25006.4006	28231.4597
ABV71579.1	UL48A	0.45007632	0.49205852	0.99945292	2041.3182	2788.68012
ABV71580.1	UL49	1.4840753	0.02610042	0.99945292	6521.67305	18243.6337
ABV71581.1	UL50	0.08929008	0.89139864	0.99945292	4674.24471	4972.68084
ABV71582.1	UL51	-0.2648164	0.6858006	0.99945292	2125.37644	1768.95624
ABV71583.1	UL52	0.41594763	0.52510838	0.99945292	9369.883	12501.0701
ABV71584.1	UL53	-0.1323504	0.83967935	0.99945292	3160.85944	2883.78842
ABV71585.1	UL54	0.1601134	0.80647097	0.99945292	18092.5681	20216.1857
ABV71586.1	UL55	-0.1474386	0.82154868	0.99945292	10280.0367	9281.34868
ABV71587.1	UL56	0.59726222	0.36229237	0.99945292	62021.3382	93828.5571
ABV71588.1	UL57	0.4584156	0.4838044	0.99945292	25524.2263	35071.1136
ABV71589.1	UL59	0.43577157	0.50588041	0.99945292	2002.74237	2708.98692
ABV71590.1	UL60	0.57717851	0.37861344	0.99945292	8389.65271	12516.776
ABV71591.1	UL69	0.58210119	0.37454809	0.99945292	17348.341	25970.9651
ABV71592.1	UL70	-0.0540523	0.9341187	0.99945292	8800.17363	8476.56375
ABV71593.1	UL71	0.28161891	0.66673014	0.99945292	9465.90143	11506.3594
ABV71594.1	UL72	0.45879223	0.4834615	0.99945292	16227.789	22303.333
ABV71595.1	UL73	0.07842644	0.90472325	0.99945292	1718.05614	1814.03816
ABV71596.1	UL74	0.03365117	0.95898594	0.99945292	6297.7985	6446.42324
ABV71597.1	UL75	-0.0678972	0.91728796	0.99945292	8793.26704	8389.01758
ABV71598.1	UL76	-0.4638675	0.47910815	0.99945292	1992.12981	1444.36644

ABV71599.1	UL77	0.34386878	0.59911817	0.99945292	13244.8164	16809.7379
ABV71600.1	UL78	0.23939243	0.71433953	0.99945292	4222.95807	4985.18744
ABV71601.1	UL79	-0.0711519	0.9134745	0.99945292	2631.74734	2505.10032
ABV71602.1	UL80	-0.0292556	0.9644491	0.99945292	2715.80558	2661.28734
ABV71604.1	UL82	-0.0300039	0.96342367	0.99945292	8357.14121	8185.13098
ABV71605.1	UL83	0.2945523	0.65242514	0.99945292	10487.9082	12863.4705
ABV71606.1	UL84	-0.0477082	0.94185616	0.99945292	6947.86013	6721.85921
ABV71607.1	UL85	-0.0054214	0.9934912	0.99945292	3709.17523	3695.26292
ABV71608.1	UL86	0.1571193	0.81001109	0.99945292	24290.1368	27084.9247
ABV71609.1	UL87	0.08667456	0.89450506	0.99945292	10864.7384	11537.4805
ABV71610.1	UL88	0.1839204	0.77849294	0.99945292	5060.33988	5748.38066
ABV71611.1	UL89	-0.2515926	0.70044044	0.99945292	7442.60775	6251.55302
ABV71611.1	UL89	-0.2515926	0.70044044	0.99945292	7442.60775	6251.55302
ABV71612.1	UL90	-0.4334116	0.50937414	0.99945292	630.015684	466.525126
ABV71613.1	UL91	-0.3195474	0.62582983	0.99945292	1177.82611	943.811741
ABV71614.1	UL92	-0.3369261	0.6068768	0.99945292	2048.05633	1621.49475
ABV71615.1	UL93	-0.0380313	0.95364702	0.99945292	6878.12043	6699.17283
ABV71616.1	UL94	0.13342276	0.83827813	0.99945292	7017.43139	7697.37372
ABV71617.1	UL95	0.39599325	0.5451784	0.99945292	4897.95081	6444.96899
ABV71618.1	UL96	-0.4563135	0.48689032	0.99945292	877.13681	639.290665
ABV71619.1	UL97	0.37031156	0.57136792	0.99945292	38941.0336	50336.4323
ABV71620.1	UL98	0.33006536	0.61391296	0.99945292	4939.5588	6209.37962
ABV71621.1	UL99	0.31601403	0.62910975	0.99945292	3473.00357	4323.50124
ABV71622.1	UL100	-0.3386341	0.60485598	0.99945292	3878.80779	3067.31545
ABV71623.1	UL102	-0.038011	0.95367009	0.99945292	7040.17259	6857.10496
ABV71624.1	UL103	-0.4796015	0.46435713	0.99945292	2117.79604	1518.82432
ABV71625.1	UL104	-0.2848252	0.66323582	0.99945292	6367.36975	5226.59383
ABV71626.1	UL105	0.29255183	0.65461549	0.99945292	13349.9313	16351.0658
ABV71627.1	UL112	0.10454772	0.87291559	0.99945292	11267.6789	12114.529
ABV71627.1	UL112	0.10454772	0.87291559	0.99945292	11267.6789	12114.529
ABV71628.1	UL114	0.09528055	0.88424481	0.99945292	2543.47776	2717.13075
ABV71629.1	UL115	-0.2991991	0.64763505	0.99945292	2218.86807	1803.27667
ABV71630.1	UL116	0.03415697	0.95841071	0.99945292	3858.76184	3951.21187
ABV71631.1	UL117	-0.2447928	0.70822097	0.99945292	4468.90002	3771.4659
ABV71632.1	UL119	-0.1839499	0.77852288	0.99945292	3894.47396	3428.26163
ABV71632.1	UL119	-0.1839499	0.77852288	0.99945292	3894.47396	3428.26163
ABV71633.1	UL120	-0.3052302	0.64107642	0.99945292	2006.78525	1624.11241
ABV71634.1	UL121	0.65135363	0.32089595	0.99945292	4655.04102	7311.41434
ABV71635.1	UL122	-0.1536427	0.81420492	0.99945292	7018.77901	6309.72324
ABV71635.1	UL122	-0.1536427	0.81420492	0.99945292	7018.77901	6309.72324

ABV71635.1	UL122	-0.1536427	0.81420492	0.99945292	7018.77901	6309.72324
ABV71636.1	UL123	-0.0496818	0.93944127	0.99945292	8371.96511	8088.56842
ABV71636.1	UL123	-0.0496818	0.93944127	0.99945292	8371.96511	8088.56842
ABV71636.1	UL123	-0.0496818	0.93944127	0.99945292	8371.96511	8088.56842
ABV71637.1	UL124	0.05642721	0.93132087	0.99945292	2975.39225	3094.07374
ABV71638.1	UL127	-0.3800662	0.56204915	0.99945292	1202.92567	924.324719
ABV71639.1	UL128	-0.2238923	0.7325445	0.99945292	1237.79552	1059.86132
ABV71639.1	UL128	-0.2238923	0.7325445	0.99945292	1237.79552	1059.86132
ABV71639.1	UL128	-0.2238923	0.7325445	0.99945292	1237.79552	1059.86132
ABV71640.1	UL130	-0.440646	0.5012667	0.99945292	2163.61536	1594.15475
ABV71641.1	UL131A	-0.504195	0.4425908	0.99945292	839.90861	592.17279
ABV71641.1	UL131A	-0.504195	0.4425908	0.99945292	839.90861	592.17279
ABV71642.1	UL132	0.07603139	0.90748049	0.99945292	3742.02364	3944.52229
ABV71643.1	UL148	-0.4277006	0.51384986	0.99945292	2321.11928	1725.61943
ABV71644.1	UL147A	-0.4286444	0.51365638	0.99945292	836.202636	621.257898
ABV71645.1	UL147	-0.4318254	0.51007603	0.99945292	1417.53529	1050.84494
ABV71646.1	UL146	-0.0364506	0.9557882	0.99945292	1647.47417	1606.37049
ABV71647.1	UL145	-0.367125	0.57526494	0.99945292	1492.6655	1157.29643
ABV71648.1	UL144	-0.1532495	0.81484482	0.99945292	2424.88657	2180.51052
ABV71649.1	UL142	-0.2060127	0.75279889	0.99945292	3685.08639	3194.70822
ABV71650.1	truncated UL141	-0.3695639	0.57352567	0.99945292	648.208651	501.718106
ABV71651.1	UL140	-0.3328304	0.61144315	0.99945292	1403.3852	1114.25047
ABV71652.1	UL139	-0.2765652	0.6728289	0.99945292	1487.10654	1227.68239
ABV71653.1	UL138	-0.3296691	0.61520049	0.99945292	873.599289	695.134071
ABV71654.1	UL137	0.59479065	0.3662836	0.99945292	306.585173	463.034913
ABV71655.1	UL136	0.14697631	0.82224047	0.99945292	2624.16693	2905.60225
ABV71656.1	UL135	0.0910527	0.88933987	0.99945292	2734.167	2912.29182
ABV71657.1	UL134	-0.4663303	0.47815733	0.99945292	526.416849	381.014909
ABV71658.1	UL133	-0.0149264	0.9819115	0.99945292	3088.92984	3057.13566
ABV71659.1	UL148A	0.0125412	0.98515138	0.99945292	828.95914	836.196843
ABV71660.1	UL148B	-0.5332095	0.41728987	0.99945292	638.943714	441.511933
ABV71661.1	UL148C	-0.3534935	0.59005272	0.99945292	779.265389	609.914706
ABV71662.1	UL148D	-0.9656843	0.14630708	0.99945292	291.424367	149.206602
ABV71663.1	UL149	-0.2650892	0.72537595	0.99945292	18.5298731	15.415107
ABV71664.1	UL150	0.76136842	0.24656959	0.99945292	11210.2363	19002.4642
ABV71665.1	US2	0.40046672	0.54132201	0.99945292	826.095432	1090.40068

Supplemental Document 2

CMV infected Kasumi-3 myeloid progenitor cell cultures in approximately 32 T-25 Falcon flasks

20 x 20 Beam	Beam Uniformity (± 2.5%)	Irr Sample Quantity 10 T-25 flasks ~~8M~~ KAS-3 CMV cells	Ion & Energy: 150 MeV Proto ~~Dose Rate ~0.1cGy/min or 6 cGy/hr~~	Files: skm_p150_1.dat 2 through dat 5

1 Expt #1: 150 MeV/n Proton Dose Response

sham - 0.0 *walk in walk out (no radiation) controls*

Kasumi-3 myeloid progenitor cells (ATCC CRL-2725) *latently infected* ***with CMV strain TB40/E.***

Entry #	Flask #'s	Desired Total Dose Gy	Dose cGy	Start	End	Deliv. Dose cGy	Dose Rate cGy/min	Irr Time min	~~Beam Running Time min~~	Comments & File
1	1,2	0.10	10.00	13:53:18	13:53:29	10.0145	22.2544	0.45	/	OK
2	3,4	0.50	50.00	13:59:09	14:00:06	50.0010	56.8632	1.02	/	OK
3	5,6	1.00	100.00	14:05:02	14:06:43	100.0090	60.3312	1.89	/	OK
4	7, 8	2.00	200.00	14:11:24	14:14:42	200.008	65.623	3.24	/	OK
5	Sham No Radiation	0.00	No Radiation	Walk in (hallway incub.)	Walk Out left out 3 1/4 min at RT				/	No Rad OK
~~6~~										

END

*LDR= Low Dose Rate

15 minutes entry time total

Setup: 13:30

Start Exposure: 13:50

End: 14:15

all good. No beam problems or delays

*** Center Turntable at Position 2 Only ***<br>Time of exposure, in minutes							
Dose (Gy)	Dose (cGy)	No Attenuation	2X	5X	10X	50X	100X
0	0	0.00	0.00	0.00	0.00	0.00	0.00
0.005	0.5	0.00	0.01	0.02	0.04	0.17	0.35
0.01	1	0.01	0.02	0.04	0.08	0.34	0.70
0.025	2.5	0.02	0.04	0.09	0.19	0.85	1.76
0.05	5	0.04	0.08	0.19	0.38	1.70	3.52
0.1	10	0.08	0.16	0.38	0.76	3.40	7.04
0.2	20	0.15	0.33	0.76	1.53	6.80	14.07
0.25	25	0.19	0.41	0.95	1.91	8.49	17.59
0.5	50	0.38	0.81	1.89	3.81	16.99	35.18
0.75	75	0.57	1.22	2.84	5.72	25.48	52.76
1	100	0.75	1.63	3.78	7.63	33.98	70.35
1.25	125	0.94	2.03	4.73	9.53	42.47	87.94
1.5	150	1.13	2.44	5.67	11.44	50.97	105.53
1.75	175	1.32	2.85	6.62	13.35	59.46	123.12
2	200	1.51	3.25	7.56	15.25	67.96	140.70
2.5	250	1.89	4.07	9.45	19.07	84.95	175.88
3	300	2.26	4.88	11.35	22.88	101.93	211.06
4	400	3.02	6.51	15.13	30.51	135.91	281.41
5	500	3.77	8.14	18.91	38.13	169.89	351.76
6	600	4.53	9.76	22.69	45.76	203.87	422.11
7.5	750	5.66	12.21	28.36	57.20	254.84	527.64
8	800	6.04	13.02	30.25	61.01	271.83	562.82
10	1000	7.55	16.27	37.82	76.27	339.78	703.52
12	1200	9.06	19.53	45.38	91.52	407.74	844.23
Dose rate (cGy/min)		132.52	61.45	26.44	13.11	2.94	1.42

NSRL 21B P.I.: Satish Mehta 281-483-5459 satish.k.mehta@nasa.go Wednesday May 26, 2021

Doug Diak 831-875-4139 douglass.m.diak@nasa.gov

Simple GCR Sim 20 x 20 Beam *(holder: small foam steps)* Access: Paula, Deborah, Adam, Jessica, Mike

Simple GCR sim		*Beam Size 60 x 60*	*Uniformity* (± 2.5 %)*					*# Samples*	*Number of Exposures 4*
Entry #	*Ion Species*	*Dose cGy*	*Start*	*End*	*deliv. Dose*	*Dose Rate cGy/min*	*Irr Time Min*		*Comments & File*
**1**	*sGCR 200*	*SM sGCR 200*							
[illegible]	p1000	70.00	13:42:44	13:44:10	70.0066	49.4320	1.60	2-T25	OK
1.0%	si600	2.00	13:46:29	one spill	2.0011	20.0030	0.30	Flasks	OK
[illegible]	he250	36.00	13:49:35	14:06:35	35.9995	2.1558	16.80	↓	OK
6.0%	o350	12.00	14:08:43	14:10:39	12.0100	5.8231	2.30		OK
1.0%	fe600	2.00	14:12:43	14:14:37	1.99870	0.9990	2.10		OK
[illegible]	p250	78.00	14:16:19	14:18:52	78.0247	30.7616	2.80		OK
100.0%	sGCR	200.00	*0:36*	hr:min	200.0406	*cGy Total*	25.90 min		*sGCR 200 complete all good*
**2**	*sGCR 100*	*SM sGCR 100*							
[illegible]	p1000	35.00	14:26:29	14:32:08	35.0015	6.0957	6.00	2-T25	OK
1.0%	si600	1.00	14:34:32	14:35:43	0.9995	1.3000	0.30	Flasks	OK
[illegible]	he250	18.00	14:37:48	14:46:54	17.9997	1.9565	9.30	↓	OK
6.0%	o350	6.00	14:49:32	14:51:08	6.0014	3.7296	1.80		OK
1.0%	fe600	1.00	14:53:11	14:54:14	0.99930	0.9086	1.20		OK
[illegible]	p250	39.00	14:55:44	14:56:37	39.0084	58.9676	0.90		OK
100.0%	sGCR	100.00	*0:30*	hr:min	100.0098	*cGy Total*	19.50 min		*sGCR 100 complete all good*
**3**	*sGCR 50*	*SM sGCR 50*							
[illegible]	p1000	17.50	15:03:02	15:05:53	17.5007	6.1760	3.10	2-T25	OK
1.0%	si600	0.50	15:08:23	15:09:05	0.4987	0.6232	0.90	Flasks	OK
[illegible]	he250	9.00	15:11:30	15:16:45	8.9993	1.7305	5.30	↓	OK
6.0%	o350	3.00	15:18:53	15:20:24	3.0000	1.9996	1.70		OK
1.0%	fe600	0.50	15:22:23	15:23:14	0.4994	0.5550	1.00		OK
[illegible]	p250	19.50	15:24:53	15:27:12	19.508	8.3556	2.60		OK
100.0%	sGCR	50.00	*0:24*	hr:min	50.0061	*cGy Total*	14.60 min		*sGCR 50 complete all good*
**4**	*sGCR 10*	*SM sGCR 10*							
[illegible]	p1000	3.50	15:34:06	15:34:44	3.5001	5.8344	0.80	2-T25	OK
1.0%	si600	0.10	15:37:32	15:37:43	0.0986	0.3288	0.41	Flasks	OK
[illegible]	he250	1.80	15:40:36	15:41:32	1.7988	1.9978	1.00	↓	OK
6.0%	o350	0.60	15:44:00	15:44:48	0.5999	0.6664	1.00		OK
1.0%	fe600	0.10	15:46:52	15:47:02	0.1003	0.5014	0.40		OK
[illegible]	p250	3.90	15:48:49	15:49:15	3.9013	8.6008	0.70		OK
100.0%	sGCR	10.00	*0:15*	hr:min	9.9990	*cGy Total*	4.31 min		
**0**	*No Radiation Sham*		*No Radiation Control*						*no radiation OK*
			Total GCR time	2:06 *hh:mm*					

Comments: ***All good, no problems.*** *GCR runs all good.*

10 cGy & 50 cGy sets placed in separte incubator from 100cGy & 200 cGy sets.

Unirradiated controls kept at room temperature for 30 minutes (same time as 100 cGy GCR) and returned their own incubator so as not to be in proximity with any irradiated samples.

Set Up: 7:30
Start: 13:42
End: 15:49

20 x 20 Bean	Beam Uniform ity (±	*Ions & Energies: 600 MeV/n Iron and 600 MeV/n Carbon*<br>**Irr Sample Quantity: 10 T-25 flasks Kas-3 CMV cells**	**Files:**<br>skm_fe600_1.dat 2 thru dat 5

Expt #1: 600MeV/n Carbon Dose Response ***Kasumi-3 myeloid progenitor cells (ATCC CRL-2725)***

latently infected with ___strain _____.

Entry #	Flask #'s	Desired Total Dose Gy	Dose cGy	Start	End	Deliv. Dose cGy	Dose Rate cGy/min	Irr Time min	File: skm_c600_1 to dat 4
1	1,2	0.10	10.00	11:09:29	11:15:32	10.0044	1.7208	6.10	OK c600_mehta.dat-3
2	3,4	0.50	50.00	11:22:48	11:41:50	50.0062	2.4890	19.60	OK c600_mehta.dat-4
3	5,6	1.00	100.00	11:48:43	12:13:40	100.0055	4.0243	25.10	OK dat-5
4	7, 8	2.00	200.00	12.19:30	13:01:23	200.0072	4.7162	42.70	OK dat-6
5	Sham No Radiation	0.00	No Radiatio	*samples left in cell room on cart from 12:19:30 to 13:03*					No Rad OK

Expt #2: 600MeV/n IRON Dose Response ***Kasumi-3 myeloid progenitor cells (ATCC CRL-2725)***

sham - 0.0 *walk in walk out (no radiation) controls* latently infected with ___strain _____.

Entry #	Flask #'s	Desired Total Dose Gy	Dose cGy	Start	End	Dose cGy	Dose Rate cGy/min	Irr Time min	File: skm_fe600_1 to dat 4
1	1,2	2.00	200.00	13:24:00	14:21:22	200.0041	3.4891	57.50	OK fe600_mehta.dat-4
2	3,4	1.00	100.00	14:32:18	14:51:15	100.0041	5.2409	19.30	OK dat 5
3	5,6	0.50	50.00	14:57:22	15:06:50	50.0036	5.2438	9.80	OK dat 6
4	7, 8	0.10	10.00	15:12:21	15:14:03	10.0054	5.6031	2.00	OK dat 7
5	Sham No Radiation	0.00	No Radiatio	*samples left in cell room on cart from 12:19:30 to 13:03*					No Rad

Setup: **8:30**
Start: **11:09**
End: **15:14**

Comments: Bit of a late start w/ EBIS switch repair, irradiations all good

All good no beam problems or delays.

Row										
8	20 x 20 Bea	Beam Uniformit y (±	Ions & Energies: 600 MeV/n Iron and 600 MeV/n Carbon						Files:	
9			Irr Sample Quantity: 10 T-25 flasks Kas-3 CMV cells						skm_fe600_1.dat 2 thru dat 5	
10										
11	***Expt #1: 600MeV/n Carbon Dose Response***					*Kasumi-3 myeloid progenitor cells (ATCC CRL-2725)*				
12						*latently infected with ___strain _____.*				

Row	Entry #	Flask #'s	Desired Total Dose Gy	Dose cGy	Start	End	Deliv. Dose cGy	Dose Rate cGy/min	Irr Time min	File: skm_c600_1 to dat 4
13										
14	1	1,2	0.10	10.00	11:09:29	11:15:32	10.0044	1.7208	6.10	OK c600_mehta.dat-3
15	2	3,4	0.50	50.00	11:22:48	11:41:50	50.0062	2.4890	19.60	OK c600_mehta.dat-4
16	3	5,6	1.00	100.00	11:48:43	12:13:40	100.0055	4.0243	25.10	OK dat-5
17	4	7, 8	2.00	200.00	12.19:30	13:01:23	200.0072	4.7162	42.70	OK dat-6
18	5	Sham No Radiation	0.00	No Radiation	*samples left in cell room on cart from 12:19:30 to 13:03*					No Rad
19										
20										

Row				
21	***Expt #2: 600MeV/n IRON Dose Response***		*Kasumi-3 myeloid progenitor cells (ATCC CRL-2725)*	
22	*sham - 0.0*	*walk in walk out (no radiation) controls*	latently infected with ___strain _____.	

Row	Entry #	Flask #'s	Desired Total Dose Gy	Dose cGy	Start	End	Dose cGy	Dose Rate cGy/min	Irr Time min	File: skm_fe600_1 to dat 4
23										
24	1	1,2	2.00	200.00	13:24:00	14:21:22	200.0041	3.4891	57.50	OK fe600_mehta.dat-4
25	2	3,4	1.00	100.00	14:32:18	14:51:15	100.0041	5.2409	19.30	OK dat 5
26	3	5,6	0.50	50.00	14:57:22	15:06:50	50.0036	5.2438	9.80	OK dat 6

... Mehta fe600 c600 5-17-23 | Sheet2

[illegible] Names: P. Bennett, M. Sivertz

CMV infected Kasumi-3 myeloid progenitor cell cultures in approximately 10 T-25 Falcon flasks

20 x 20 Beam	Beam Uniformity (± 2.5%)	Ions & Energies: 600 MeV/n Iron and 600 MeV/n Carbon<br>Irr Sample Quantity: 10 T-25 flasks Kas-3 CMV cells	Files:<br>skm_fe600_1.dat 2 thru dat 5

Expt #1: 600MeV/n Carbon Dose Response *Kasumi-3 myeloid progenitor cells (ATCC CRL-2725) latently infected with ___strain _____.*

Entry #	Flask #'s	Desired Total Dose Gy	Dose cGy	Start	End	Deliv. Dose cGy	Dose Rate cGy/min	Irr Time min	File: skm_c600_1 to dat 4
1	1,2	0.10	10.00	11:09:29	11:15:32	10.0044	1.7208	6.10	ok c600-mehta.dat-3
2	3,4	0.50	50.00	11:22:48	11:41:50	50.0062	2.5890	19.6	OK dat-4
3	5,6	1.00	100.00	11:48:43	12:13:40	100.0055	4.0243	25.1	OK dat-5
4	7, 8	2.00	200.00	12:19:30	13:01:23	200.0072	4.7162	42.7	OK dat-6
5	Sham No Radiation	0.00	No Radiation	control flasks left on cart @ RT in cell room 1249 - 13:03					

Expt #2: 600MeV/n IRON Dose Response *Kasumi-3 myeloid progenitor cells (ATCC CRL-2725)*

sham - 0.0 walk in walk out (no radiation) controls latently infected with ___strain _____.

Entry #	Flask #'s	Desired Total Dose Gy	Dose cGy	Start	End	Dose cGy	Dose Rate cGy/min	Irr Time min	File: skm_fe600_1 to dat 4
1	1,2	2.0	200	13:24:00	14:21:32	200.0041	3.4891	57.50	OK fe600_mehta.dat 4
2	3,4	1.0	100	14:32:18	14:51:15	100.0041	5.2409	19.30	OK fe600-mehta.dat 5
3	5,6	0.5	50	14:57:22	15:06:50	50.0036	5.2438	9.80	ok fe-600mehta.dat 6
4	7, 8	0.1	10	15:12:21	15:14:03	10.0084	5.604	2.00	OK fe-600
5	Sham No Radiation	0.00	No Radiation						

Setup: 8:30
Start: 11:09
End: 15:14

Comments: Slight delay. Switch repair in CB15
